# Wild recognition: conducting the mark test for mirror self-recognition on wild baboons

**DOI:** 10.1098/rspb.2024.1933

**Published:** 2025-01-22

**Authors:** Esa A. Ahmad, Helen Reiderman, Elise Huchard, Axelle Delaunay, Vittoria Roatti, Guy Cowlishaw, Alecia Carter

**Affiliations:** ^1^ Department of Anthropology, University College London, London, UK; ^2^ Institut des Sciences de L'Evolution de Montpellier, UMR 5554, CNRS, Université de Montpellier, Montpellier, France; ^3^ Gobabeb Namib Research Institute, Walvis Bay, Namibia; ^4^ Department of Biology, University of Turku, Turku, Finland; ^5^ Institute of Zoology, Zoological Society of London, London, UK

**Keywords:** baboon, mark test, mirror self-recognition, *Papio ursinus*, self-awareness

## Abstract

The distribution of self-awareness across species is important to understand, not only as a matter of scientific interest but also because of its implications for the ethical standing of non-human animals. The prevailing methodology for determining self-awareness is to test for visual self-recognition using mirror-image stimulation and a ‘mark test’. However, most studies have involved very small sample sizes, omitted a control condition and been conducted on captive animals. Here, we designed and implemented the first controlled mark test in a wild setting, conducting the mark test using a laser pointer on a large (*n* = 51 individuals, 135 mark tests) sample of wild chacma baboons (*Papio ursinus*) *in situ*. Control tests showed that baboons were interested in the mark, but this interest decreased with age, and was greater in males and towards green (cf. red) marks. However, as predicted, subjects showed no evidence of visual self-recognition, which, given the control, cannot be attributed to a lack of motivation in the mark. Our study proposes a novel, controlled mark test *in situ* and contributes to the evidence that, without extensive training, non-hominid primates are not capable of full visual self-recognition.

## 1. Introduction

Cognitive psychology explores how humans and other animals learn, process information, solve problems and experience the world. Self-awareness, a concept in this field, is ambiguous, but could be defined as ‘the capacity to become the object of your own attention’ [1]. Despite difficulties in satisfactorily defining self-awareness, efforts have been made to determine its distribution in the animal kingdom. The most-commonly used test of self-awareness is that of visual self-recognition (VSR), which is demonstrated by an individual’s ability to identify an image as oneself, particularly by manipulating parts of oneself that are usually non-visible. VSR tests generally involve mirror-image stimulation (MIS). Highly reflective surfaces in which clear reflections can be seen are rare in the environment and provide a novel stimulus to test VSR by making an animal an audience to itself. The criterion for evidence of VSR in response to MIS is the spontaneous emergence of mirror-guided self-directed behaviour [2], such as investigating body parts otherwise non-visible, accompanied by a cessation of social behaviour towards the mirror [3,4].

In addition to MIS, a widely adopted, more rigorous experimental protocol, the mark test, was devised independently by Gallup [5], as a psychologist studying primate cognition, and by Amsterdam [6], as a clinical child psychologist. The mark test involves marking a part of an individual’s body not visible without a mirror and quantifying the individual’s response to the mark when later exposed to a mirror. The simplicity and intuitiveness of the mark test protocol have made it the most widely adopted tool to date for testing for VSR. However, the mark test has been criticized as not demonstrating complex psychological self-awareness but simply demonstrating bodily self-awareness. For example, Mitchell [7–10] has argued that passing the mark test demonstrates two faculties. First, the faculty to recognize mirror correspondence, which is the relationship between objects in the mirror and objects in reality. Second, it demonstrates ‘kinaesthetic-visual matching’, which is the faculty to form a mental outline of one’s own body from proprioceptive feedback. Mitchell [7] argues that the combination of these cognitive faculties is sufficient to produce functional VSR and that the mark test demonstrates only these. At its core, to pass the mark test demonstrates: an understanding that the reflection represents one’s own body, the ability to generalize that understanding to parts of one’s body not visible without the mirror and to act according to that understanding to explore a novel stimulus.

Although it remains debated exactly what sort of self-awareness VSR represents, its ontogeny in humans and distribution across non-human animals does point to it being indicative of *something*. In humans, VSR emerges gradually; by 2 years of age, 65% of children demonstrate VSR [6]. VSR is most common in the great apes; since the Gallup study [5], VSR in chimpanzees has been well replicated using mark tests [11–15] as well as by MIS without mark tests [16]. Orangutans [17,18] and bonobos [16,19] have also shown evidence of VSR, but gorillas have produced mixed results [15–17,20–22]. Outside the great apes, there is no convincing evidence of spontaneous VSR in any other primate [16,18,23]. One initially promising finding of VSR in cotton-top tamarins [24], for example, later failed to be replicated [25]. This suggests that this faculty is homologous within the primate order and evolved in the common ancestor of the great apes. While most MIS studies have been conducted on primate species, studies have also been conducted on more distant taxa, producing debatable evidence for VSR. For example, MIS studies and mark tests have been conducted on species as diverse as Asian elephants [26,27], horses [28,29], dolphins [30,31], pigeons [32,33], corvids [34–37], cleaner wrasse [38,39], manta rays [40] and ants [41]. While some of these claims are plausible, many are weakened by lack of replication, small sample sizes and the inherent difficulty of interpreting mark-directed behaviour in species without hands.

One exception to the non-hominin primate pattern of failing VSR is worth mentioning. Chang *et al*. [42,43] used visual-somatosensory training to elicit VSR in rhesus macaques. After training, the subjects were able to pass the mark test to a similar standard as chimpanzees. While such training has been criticized, importantly in this experiment, the trained monkeys subsequently showed spontaneous, *untrained* mirror-guided self-directed behaviour of the kind expected of self-recognizing animals. The trained macaques spontaneously used mirrors to aid self-grooming of normally non-visible body parts (e.g. under the chin) and to inspect their own genitals. While these results have been criticized due to the extensive training involved [18,44], the training cannot explain the untrained, spontaneous use of the mirror and suggest that macaques have a VSR faculty that is similar in kind, although not equal in degree, to that of a chimpanzee. These findings, along with others suggesting intermediate levels of mirror understanding in some monkey species [45,46], contribute to an interpretation of VSR as a graded faculty [18]. These studies have motivated ours in demonstrating that the search for elements of VSR in monkeys can be productive.

Most MIS studies suffer from small samples, a lack of control and were conducted in captivity. In the first case, with some notable exceptions [14,47–49], the number of subjects in MIS studies rarely exceeds ten and can include as few as two subjects [50–52] or even one [20,21,53]. Given the variation in VSR ability even within self-recognizing species, evidence from small numbers of individuals should be generalized to the species level only with caution [54]. In the second case, lack of subjects’ motivation to investigate the reflection or the mark might produce false negatives in mark tests [55,56]. A visible mark condition, in which the subject can perceive the mark on its own body without reference to a mirror, can control for differences in motivation. The visible mark condition was introduced by Gallup *et al*. [57] but has since been used infrequently [15,21,22,24,25,58,59]. In the third case, the vast majority of studies have been conducted in captivity on ‘enculturated’ subjects: individual primates who have been raised in proximity to humans and gain abilities that wild counterparts lack. Given that, in the wild, most subjects would need to be captured and/or anaesthetized to apply the mark, and given the unpredictability of wild animals’ behaviour, it is perhaps understandable why few studies have been conducted in the wild. We know only two studies that have provided MIS in natural populations, and only one applied marks (to six individual Japanese macaques [42]). Perhaps surprisingly, a population of wild chimpanzees showed no signs of VSR when exposed to a large mirror over a 3 year period, suggesting that the difference between wild and captive animals needs further exploration [60].

In this study, we aimed to conduct a mark test in the wild on a non-human primate, the chacma baboon (*Papio ursinus*). Specifically, we aimed to design and implement a rigorous and practical *in situ* mark test protocol on wild animals with a large sample size that inflicts minimal stress on the subjects. Our hope is that this methodology can be replicated in the future on other wild populations of animals to further investigate the effect of enculturation on animal VSR. We chose to study wild chacma baboons because they have not yet been tested with the mark test, and papionins have been historically overlooked, with only three individual baboons of two species tested [12,50]. In addition, chacma baboons are a well-studied, highly social species that travel together in large groups and voluntarily interact with objects during experiments [61,62]. We used laser marks instead of paint or dye to mark the baboons because this methodology does not require capturing individuals and is thus non-invasive and immediately reversible. We tested individuals in their familiar social environment and subjects’ participation with the mirror was entirely voluntary.

In addition to designing a field-friendly, non-invasive mark test, we aimed to answer four specific research questions. (Q1) Are wild chacma baboons motivated to investigate marks when those marks are clearly visible on their own body without a mirror? This is a necessary control to determine whether using such marks would constitute a useful test and is investigated by including a visible mark condition. (Q2) Is there variation in interest in a visible mark? Although unrelated to VSR *per se*, it is worth investigating which factors affect baboon attention to minor but novel stimuli to understand where variation in responses to the mark test may arise. (Q3) Do baboons investigate those marks on their bodies that are only visible in a mirror (i.e. ‘pass’ the mirror test)? (Q4) If so, which variables predict their ability to do so? These last two questions relate directly to VSR and its variation in chacma baboons. We predicted that the laser mark would be a salient stimulus during the visible condition [42,43] and that responsiveness would be higher in males and younger individuals, as we have previously shown this variation in response to novel objects at this field site [61]. However, we predicted that the baboons would not show evidence of VSR through a positive mark test result. This is because current evidence indicates that monkey species do not pass the mark test without extensive training, and wild, unenculturated chimpanzees did not show evidence of VSR [60].

## 2. Methods

### (a) Study site and species

The experiment was conducted from May to October 2021 at Tsaobis Nature Park (15°45′E, 22°23′S), Namibia. We studied individuals of two habituated troops of wild chacma baboons, J and L, consisting of 55 and 65 individuals, respectively. Tsaobis is located on the edge of the Namib desert. The habitat comprises the ephemeral Swakop River and its tributaries, bordered by dense patches of woodland, which are in turn surrounded by rocky hills with scarce vegetation. The study troops are habituated to observers on foot, their members are individually recognizable and are followed, on average, for six months every year during the austral winter (range 3−9 months). Chacma baboons live in multi-female, multi-male troops that move together as a cohesive group. Males emigrate typically at adulthood (approx. 7−9 years); females are philopatric and attain adulthood at approximately 4−6 years old. Baboon society is characterized by a strict linear dominance hierarchy that is calculated using the I&SI method from dominance interactions recorded ad libitum by all members of the field team. Subjects’ linear ranks were converted to a relative rank using the formula 1 − [(1 − *r*) ÷ (1 − *n*)], where *r* is the individual’s absolute rank and *n* is the group size. Subjects for this study were chosen semi-randomly, focusing on including variation in sex, age and social dominance rank. Age was calculated to the nearest year based either on known birth dates or estimated from data on patterns of females’ ovulation or individuals’ dental eruption (see [63] for details).

### (b) Mirror exposure

**Figure 1.**
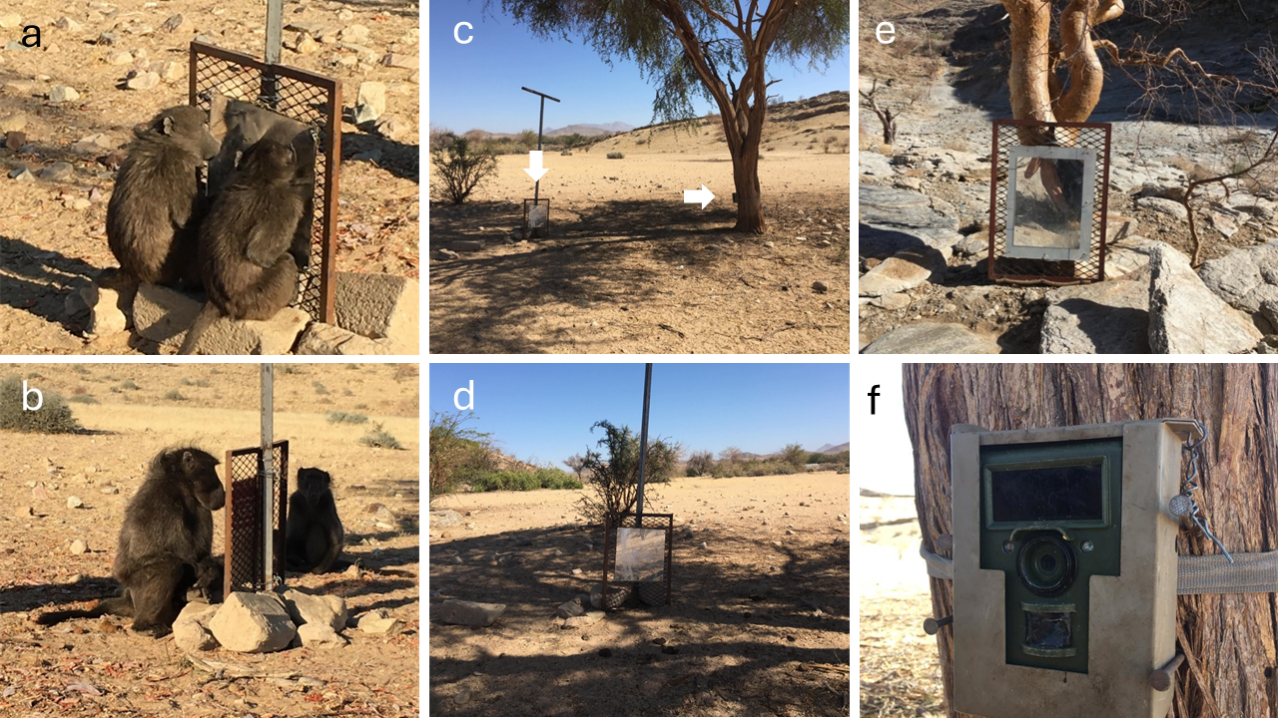
The set-up of the mirrors during mirror exposure and the mark test. (*a*) Two juvenile baboons exploring mirror 1 together. (*b*) An adult male interacting with the mirror. (*c*) Mirror 1 (vertical arrow) with the camera trap, mounted on a tree (horizontal arrow), trained on it. (*d*) Mirror 1, visited most days by the two troops. (*e*) Mirror 2, visited more frequently by J troop. (*f*) The camera trap set-up to record mirror interactions at mirror 1.

To give baboons the opportunity to learn about the reflective surface of a mirror, we provided a period of mirror exposure before the initiation of the mark test and controls. Two medium-sized (approx. 50 × 50 cm) Perspex-backed safety mirrors were placed near frequented water points. Holes were drilled through the corners of the mirrors, and the mirrors were then attached using fencing wire to a metal frame approximately 30 cm from the ground (figure 1). This height allowed an average adult female to view her face when seated in front of the mirror. The frame was fastened securely to a metal pole or a tree using fencing wire. Individuals had physical access to the mirrors from all sides and were able to explore them together with others (figure 1*a*,*b*). Mirrors were cleaned every few days to retain visibility. Mirror 1 (figure 1*c*,*d*) was in the field from mid-May until the beginning of October 2021, providing mirror exposure before the first mark tests in mid-June. Mirror 2 (figure 1*e*) was in the field for the month of September, being added because baboon movement patterns had hindered data collection at mirror 1.

To track subjects’ mirror exposure, a motion-sensor video camera trap (Bushnell Natureview Essential HD camera) was attached to a tree near mirror 1, approximately 1 m from the ground using fencing wire, nails, fabric straps and bungee cords (figure 1*f*). The camera was aimed to capture the mirror and any individual in front of it and was triggered by motion to record for 60 s during the day and 15 s at night (when baboons were not present) with a 1 s interval between videos. Video resolution was set to 1920 × 1080 px. Batteries were changed every 3 days. Mirror interactions at mirror 2 were not captured by a camera trap because of limited equipment.

### (c) Mark tests

From 19 June to 1 October 2021, individuals were subjected to the mark test, which included three test conditions: ‘visible control’, ‘non-visible control’ and ‘mirror’, described below. Tests were conducted opportunistically by one experimenter (E.A.) at a distance of 5−10 m when subjects were either resting or sitting in front of the mirror. All tests were video recorded with a handheld camera (FujiFilm FinePix XP140 digital camera). Marks were applied using two colours: a green (532 nm) and a red (635 nm) laser pointer of output power 5 mW. This output power is considered safe for commercial use by the US Food and Drug Administration [64], producing no irritation when shone directly onto the skin and being safe in the event that the laser passes briefly over the subject’s eyes (which never occurred). The experimenter was constantly vigilant to the possibility of the laser passing over the subject’s eyes and responded immediately if this became a danger. Tests lasted for no more than 90 s, and usually less.

The visible test condition was the first control, important to determine whether the subjects would investigate a mark visible on their own bodies. If subjects did not respond to a visible mark, then marks that are non-visible for the study subjects (except when using the mirror) would not be salient stimuli for VSR. In this control condition, the mark was shone on a visible part of a subject’s body while the test subject was resting in the absence of the mirror. The mark was shone on the hand or foot and removed if the subject’s attention was not on the mark (e.g. the individual was approached by another baboon or watched an interaction between others). The experimenter moved the laser slowly to ensure the subject was attending to it. The moving marks of our protocol were not entirely comparable with the static marks of the traditional protocol. However, the most relevant comparison to test for VSR is the difference between this control and the mirror test, and for this, the movement was the same. The second control, the non-visible mark condition, was used to determine whether the laser mark could be felt by a subject and to establish a baseline rate of facial touching. In this condition, the experimenter shone the mark on a part of a baboon’s body that was not visible without a mirror (i.e. on the subject’s cheek or ear) when the subject was not facing a mirror. Finally, the test condition, the mirror mark test, determined whether the subject recognized its own face in the mirror. The mark was applied to the same ‘non-visible’ parts of a subject’s body (i.e. the cheek or ear) when the subject was looking into the mirror. If the subject was not looking in the mirror, the mark was removed. As in the visible condition, the mark was moved slowly to increase its salience.

For each presentation, a video coder (E.A.) recorded the following information: the (i) test subject’s identity, age (in years), sex, relative rank and troop; (ii) test condition; (iii) number of mark-directed touches (defined below); and, as control variables, the (iv) test duration; (v) mark colour; (vi) laser exposure incurred (described below); (vii) total prior laser exposure of the subject; and (viii) total prior mirror exposure of the subject. Prior mirror exposure was calculated from the camera trap footage. Where test duration was under 2 s, tests were excluded from the final dataset since such a short test did not contribute meaningfully to an individual’s mark exposure.

A mark-directed touch was defined as a clean, precise and direct touch of the mark. Common self-directed behaviours such as self-scratching and nose-wiping were not included as mark touches. Under the visible condition, test duration included only the time during which the mark was within the subject’s field of vision. Mouthing or licking the mark in the visible condition (median = 0, mean = 0.54, range = 0–11) was not included as mark-directed behaviour in the main analysis because there was no analogous behaviour possible under the mirror condition. However, we also ran the core analysis (Q1) with mouthing included (see electronic supplementary material) and found no differences in the key findings reported here. Under the non-visible condition, test duration included all the time when the mark was shone on a subject’s body. Under the mirror condition, test duration included only the time during which the subject could view the mark on his/her body (i.e. when s/he was looking into the mirror).

A subject’s prior laser exposure at each test was calculated to control for habituation and its possible effect on touch rate. The coder recorded the cumulative length of time across all tests during which the laser point was in a subject’s field of vision. This value was independent of an individual’s time tested and included individuals’ sightings of the laser on the ground or on another individual. The total did not include exposures of under 2 s.

An individual’s total prior mirror exposure at each test was recorded in case longer exposure gave individuals a better understanding of the mirror and was more likely to demonstrate VSR. Prior mirror exposure time included all the time during which an individual remained continuously in front of the mirror after having first looked into it, which included but was not limited to time during tests. Again, periods under 2 s were not counted.

A second, independent coder (HR) coded 20% of randomly selected tests under each condition to ensure interobserver reliability. This was important because, although criteria for counting mark-directed touches were strict, personal judgement could be involved.

### (d) Statistical analysis

We first tested interobserver reliability between coders using the intraclass correlation (ICC) function in the *psych* package. Next, we fitted three statistical models to address our research questions. All statistical analyses were conducted in RStudio (v. 3.6.0) starting from the same model structure. All models included as fixed factors the following control variables: age in years, rank, sex, troop, mark colour, prior laser exposure and prior mirror exposure. Age was mean centred, and sex (0 = female, 1 = male), troop (1 = J, 2 = L) and mark colour (0 = green, 1 = red) were coded as binary variables. Prior laser exposure and prior mirror exposure were each converted into categorical variables with four possible values (zero, lower, middle and upper terciles; 0, 1, 2 and 3). Because the test duration was different for each observation, and could affect the number of possible touches, we included test duration as an offset to model the rate of touching, i.e. the number of touches over the test duration. Because there were repeated measures of individuals within and across conditions, we included individual identity as a random effect. We checked that models’ residuals were normally distributed and that model variance inflation factors were below 4.

To address Q1, whether the laser mark was a salient stimulus, we ran a model using the subset of the data in the control conditions, i.e. we compared rates of touching when the subject was in the visible and non-visible mark conditions while controlling for the variables stated above. However, these models struggled to converge, probably due to the lack of variation in the non-visible condition (non-visible non-mirror touches: median = 0, range 0−1, IQR = 0–0). Because of this, to confirm the Poisson GLMM results, we also ran a simplified generalized linear model with a binary response for touches (0 = did not touch, 1 ≥ 0 touches) with no control variables. We also visually inspected the distribution of the data to confirm the model’s summary (see §3).

To address Q2, whether there was variation in response to the visible mark, we included only observations under the visible test condition, and we did not include a control for prior mirror habituation, which was not relevant to this condition. We assessed the effects of the control variables on the rate of touching by comparing the full model to a null model without the variable of interest through an ANOVA (package: car), and we determined the effect size through the model summary.

To address Q3, whether the mirror condition elicited a significantly higher rate of touching than the non-visible condition, we ran a model using the subset of the data in the mirror and non-visible conditions. This compared the rate of touching when baboons had the mark on their face without a mirror present with the rate of touching when they could see the mark in the mirror. However, there was next to no variation in these categories of data (non-visible mirror touches: median = 0, range 0−1, IQR = 0–0; non-visible non-mirror touches: median = 0, range 0−1, IQR = 0–0). We thus concluded that there was no difference between these categories. Because of this, we did not perform an analysis to address Q4, determining what factors affected responses in the mark test.

## 3. Results

We completed 361 laser experiments across 112 individuals. This comprised 123 visible condition tests from 91 individuals, 103 non-visible condition tests from 85 individuals and 135 mirror condition tests from 51 individuals (see table 1 for a break down by sex). Across all test conditions, the 112 tested individuals experienced, on average (median), 3 tests totalling 95 s duration (range = 1–13 tests, 4−581 s). Higher numbers of tests and durations were due to opportunistic mirror tests for individuals who voluntarily sat at the mirror.

**Table 1.** Number of test instances under each test condition, broken down by sex.

	condition (*n*, percentage of tested population for each condition)		
sex	mirror	non-visible	visible
female	23 (17.04)	50 (48.54)	60 (48.78)
male	112 (82.96)	53 (51.46)	63 (51.22)
total *n*	135	103	123

In the mirror condition tests, where participation (i.e. interacting with the mirror) was entirely voluntary and therefore non-random, most (83%) subjects were male. The mean relative rank of subjects was 0.68 and the mean age (years) was 5.6. This differs from the semi-random sample for the visible and non-visible conditions, in which subjects were more equally distributed: 51% were male in both conditions, mean rank was 0.55 and 0.51, and mean age was 6.6 and 6.3, respectively. The youngest baboon tested in each condition was under six months; the oldest in the visible and non-visible conditions was 20 and the oldest in the mirror condition was 12. The greatest laser habituation that any subject had received prior to a mirror condition test was 9 min and the median was 80 s. The greatest mirror habituation received prior to a mirror condition test was 200 min and the median was 15 min.

The ICC3 between independent raters for the number of mark-directed touches was 0.99, indicating excellent interobserver reliability.

The majority (64.2%) of visible condition tests resulted in at least one touch. Mark touching was not an artefact of a few motivated individuals tested on several occasions, since this proportion remains similar (63.7%) when only the first visible condition test of each individual was included. Baboons touched the mark a median of 5 times (range = 0–57), with a median touch rate of 0.22 (0−1.12) touches per second. These values were similar (5 and 0.18, respectively) when only the first visible exposures were included.

**Figure 2.**
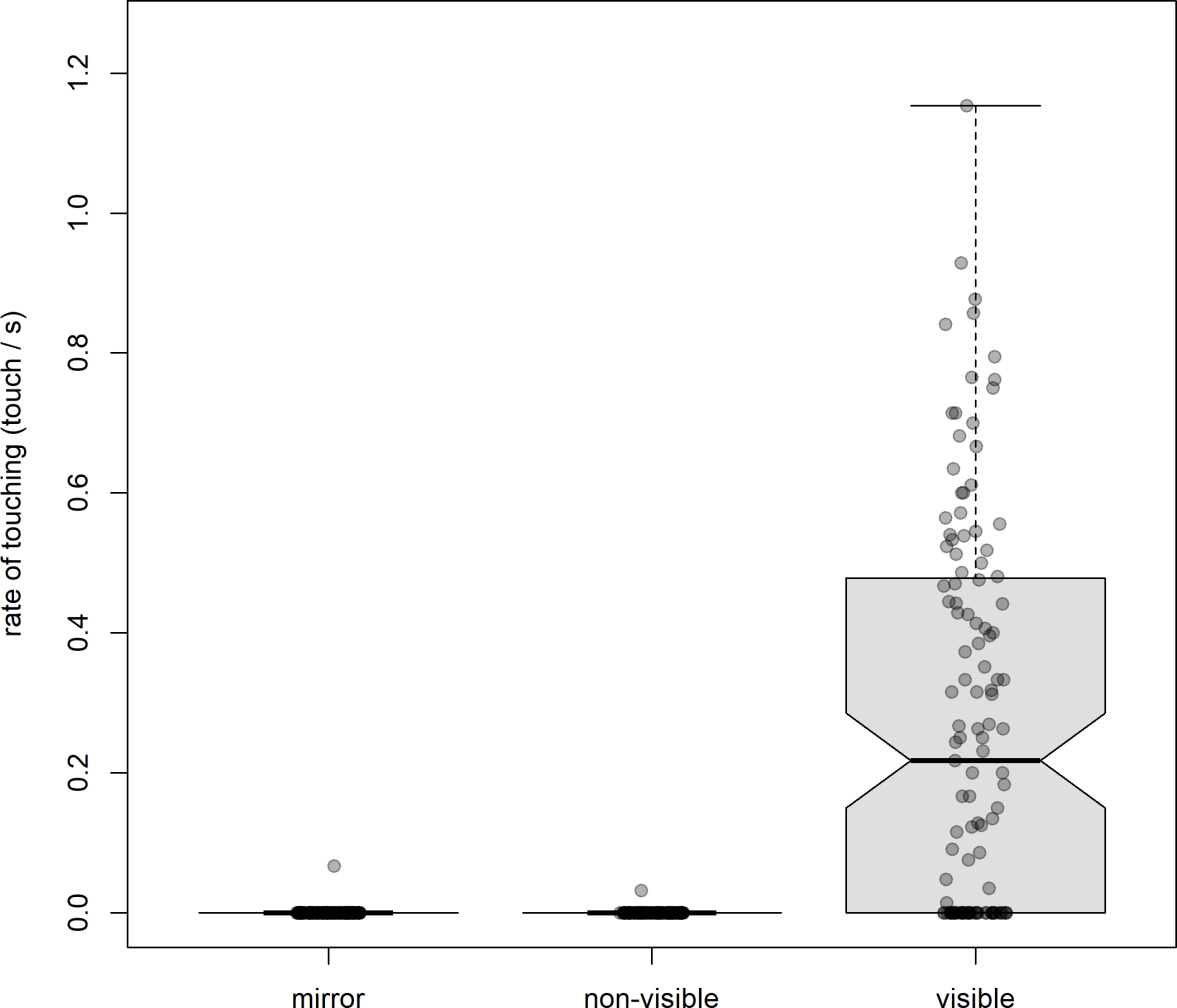
Rates of touching the mark across the test conditions. Shown are box plots indicating the median rates (notches in boxes), IQR (box ends) and minimum and maximum rates (whisker ends) at which baboons touched the laser mark under different test conditions. Overlaid are the raw data, with some observations shown as outliers. NB: data are presented as rates but were analysed as counts with or without an offset for test duration, depending on the model. See text for details.

To address Q1, whether subjects were motivated to interact with the laser mark, we fitted both a Poisson GLMM and binomial GLM to confirm the Poisson model, and found that, controlling for other variables, there was a higher rate of touching under the visible condition compared to the non-visible condition (GLMM: ± s.e. = 7.48 ± 0.98, *z* = 7.57, *p* < 0.001; GLM: β ± s.e. = 5.21 ± 1.02, *z* = 5.10, *p* < 0.001; figure 2). Overall, these models indicate that subjects found the mark visually compelling (see electronic supplementary material, videos S1 and S2).

**Figure 3.**
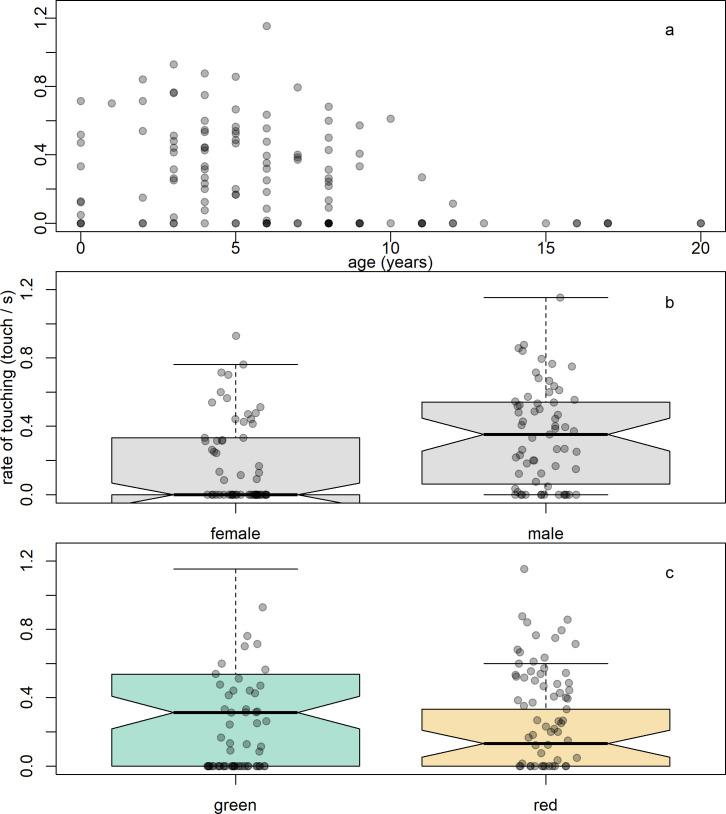
Predictors of mark-touching rate in the visible mark test condition. Shown are (*a*) a scatter plot showing the rate of touching in response to the tested individuals’ age in years; and box plots showing the effect of (*b*) sex and (*c*) laser colour on the rates of touching the mark, with the raw data shown in points.

To test Q2, determining predictors of interest in the mark in the visible condition, we ran a GLMM with the rate of touches as the response variable for the visible mark condition only. We found that the rate of touching was predicted by age, sex and mark colour. There was a negative correlation with age (table 2; figure 3*a*); males touched the marks at higher rates than females (table 2; figure 3*b*) and red marks elicited lower rates of touching than green marks (table 2; figure 3*c*). Touching rate was not predicted by troop, rank or prior laser exposure (table 2).

**Table 2.** Summary of predictors of mark touching in the visible test condition.

predictor	*β*	s.e.	log likelihood^a^	χ^2^	*p*
age	−0.21	0.04	−401.97	28.32	<0.001
relative rank	0.07	0.53	−387.82	0.02	0.89
sex^b^	0.77	0.29	−391.30	6.97	0.008
troop^c^	−0.23	0.25	−388.23	0.83	0.36
laser colour^d^	−0.63	0.13	−399.73	23.83	<0.001
prior exposure	0.08	0.06	−388.58	1.54	0.21

Shown are the effect size (β) and s.e. of the predictors from the full model and the log likelihood, χ^2^-test statistic and *p*-value from an ANOVA comparing the full model to a null model without the predictor of interest.

^a^ Full model log-likelihood = −387.81.

^b^ Reference = female.

^c^ Reference = J.

^d^ Reference = green.

To test whether baboons showed evidence of mirror self-recognition (Q3), we aimed to test whether there was a difference in the number of touches when baboons were tested on non-visible parts of the body when in front of a mirror and when not. Only one non-visible condition (of 103, 0.97%) and one mirror condition (of 135, 0.74%) test elicited touching. It was not possible to conduct a statistical test on these data. However, since the mirror condition did not elicit a different rate of touching from the non-visible condition, we conclude that the criteria for VSR were not fulfilled (see electronic supplementary material, video S3).

Since no evidence of VSR was found, we could not investigate Q4: which variables predict subjects passing the mark test?

### (a) Qualitative observations

While we did not systematically collect data other than mark-directed touching, we observed mirror and mark-related behaviours that cannot be statistically analysed. We describe these anecdotal observations here. We observed several mirror-related behaviours that included startling at the mirror, reaching behind the mirror while looking into it, threatening and shaking the mirror (which waned rapidly) and affiliative facial expressions towards the mirror. Baboons would sometimes sit extremely close to the mirror while directing affiliative expressions towards their reflections. These responses to the mirror diminished over time.

Our observations suggest that the baboons eventually understood the mirror’s reflective surface, although not their own reflection within it. We observed baboons sitting at an angle in front of the mirror, peering not at their own reflection but at that of the environment, and intermittently turning around as though comparing the images. Baboons would often startle at the far approach of a more dominant individual while looking in the mirror and check over their shoulder to confirm their approach in it. It thus seemed that the baboons were able to understand what the mirror was showing.

We also observed notable mark-related behaviours beyond mark-directed touching. Baboons sometimes startled upon seeing the mark. Under the visible condition, baboons would often mouth at and lick the mark rather than touching it manually (see electronic supplementary material, video S1). Obvious interest was sometimes directed towards the mark when it was seen on the ground as the experimenter tracked it towards a subject. On one occasion, a non-visible condition test was interrupted by a passing infant who approached the test subject and began to groom the spot where the mark had been a moment before (the experimenter having immediately stopped the test), before giving up and moving on (see electronic supplementary material, video S2). In contrast, some baboons paid no attention to a mark being shone on the fur of an individual whom they were grooming.

## 4. Discussion

MIS studies have generally been conducted on very small samples of captive animals. In this study, we aimed to develop a practical and replicable mark test protocol for use on wild primates, and to apply this to a large sample of individuals. As predicted, the baboons expressed no mark exploration when marks were visible only in the mirror and thus failed to meet the criteria for VSR. The inclusion of a visible control condition reduces the probability that the failure to show VSR was attributable to a lack of motivation—baboons touched the mark when it was visible to them. Our sample also encompassed a wide variety of individuals, including individuals of both sexes, ranging in age from under 6 months to 12 years of age, and ranged in rank from lowest (0) to highest (1) of their respective troops. While the absence of evidence is not evidence of absence, we argue that this negative result is relatively robust.

Our prediction that baboons do not show evidence of VSR was supported and agrees with the findings of the wider monkey VSR literature. No spontaneous self-directed behaviours, such as using the mirror to inspect their own genitals, of the kind observed in some great ape species [12,18,20] and mirror-trained rhesus macaques [38,39], were observed. However, although monkeys do not show VSR, data suggest they can show an intermediate form of self–other distinction. For example, brown capuchins (*Cebus apella*), when shown a live (unfamiliar) conspecific ‘stranger’ and a mirror, treated the mirror image differently from the stranger, showing friendlier behaviour and less anxiety towards the mirror image [45], and similar ‘intermediate’ behaviours implying a partial mirror understanding have been observed in spider-monkeys [46]. While we did not collect data on baboons’ responses to individual strangers as a comparison (as it would be unethical to experimentally introduce an unfamiliar baboon in the field), our observations of individuals’ mirror-directed behaviours seem to indicate something similar. For example, adult males occasionally directed affiliative gestures at very close range towards their own reflections. If two adult males were interacting with each other, it would be improbable that they would place their faces so close to each other and make affiliative expressions, so this behaviour towards the mirror seems to indicate a more nuanced understanding of the reflection than simply ‘an unknown conspecific’. Additionally, baboons would frequently sit very close to the mirror making eye contact with their reflection, not acting in an affiliative or aggressive fashion, which is similar to the response of capuchins to mirror images [45]. Future studies could investigate these observations in more detail.

The baboons also demonstrated behaviours that indicated they understood the reflective property of the mirror, as has been seen in other monkey species [45,65]. For example, baboons would ‘regard behind’ themselves when sitting in front of the mirror, suggesting that individuals explored the correspondence between the environment and its reflection. Similarly, observations of baboons startling at the approach of a dominant individual visible in the mirror, before checking behind to confirm, provide further evidence of an understanding of mirror correspondence. These behaviours have been noted in the two other wild MIS studies [49,60,66]. While this does not translate to VSR, it indicates some kind of mirror understanding. Of course, auditory or other cues cannot be ruled out, but the ability of some monkeys to use mirrors to understand their surroundings is well corroborated in captivity [55,67,68]. A future study might formally investigate this by implementing the first mirror-mediated search task in the wild. These observations, along with those in capuchins [59], spider-monkeys [46] and rhesus macaques [38,39], may strengthen the gradualist interpretation by demonstrating that certain monkey species possess intermediate mirror understanding even without full, spontaneous VSR.

One possible explanation for the lack of VSR is the fascination that subjects showed towards the mirror. Although habituation time was provided, it was possible that the subjects undergoing the mirror condition were distracted by the mirror itself. If so, we would expect a higher threshold for self-directed behaviour of any kind, since the mirror itself might remain an overpoweringly interesting stimulus. If wild primates habituate less rapidly to novel objects like the mirror because their exposure is intermittent, this could explain why wild chimpanzees failed to show self-directed behaviours towards their reflections [60]. In this case, the visible condition alone would not be sufficient to prove motivation, and future studies might include a ‘mirror-only’ condition to establish a baseline for mirror-directed interest and behaviour to differentiate interest in the mirror from interest in the mark.

Three variables predicted the level of individuals’ interest in the laser marks, as measured by touch rate: laser colour, age and sex. In the first case, red marks elicited lower touch rates than green marks. Although baboons have trichromatic vision and can certainly differentiate red from green [69], the discrepancy in touch rates is probably not indicative of a colour preference but rather that red lasers appeared less bright than green lasers for the same output power. Future studies employing this methodology could adjust the output power of the lasers to create equivalent brightness. In the second case, as predicted, older individuals performed lower rates of mark-directed touching under the visible condition. Researchers have found similar correlations of age with mirror interest and mark test performance [66,70], and this finding may be related since it describes the association of age with decreased motivation to explore novel stimuli, as has been shown in this population [61]. In the third case, and in a similar vein, males were more likely to touch the visible mark. As for age, this supports previous research in this population and other primate species (vervet monkeys *Chlorocebus aethiops*), demonstrating a decreased motivation for females to explore novel stimuli [62,71].

While not formally analysed, we noticed that individual variation played a role in the variation in mark-directed touching under the visible condition. Individual variation in mark-directed behaviour could be a result of personality differences, and in particular, individuals’ responses to novel stimuli. Such differences in personality could arise through genetic variation. For example, Mahovetz *et al.* [48] found that gene polymorphism correlated with mirror interactions in chimpanzees. Similarly, the early life environment can also affect personality, such as a mother’s maternal style, which influences behaviour in relation to exploration [72], and could thus affect baboons’ reactions to stimuli like the laser mark.

This study developed a methodology for non-invasive and efficient testing of large numbers of animals *in situ*. The use of laser pointers as easily applicable, no-contact marks is practical in a field setting from the researcher’s perspective, but is also useful in reducing interference with the animals. However, one limitation is that we did not test whether the laser was visible from different angles in the mirror on the baboons’ fur. Future studies could test whether the laser attenuates and becomes less visible in the mirror’s reflection. Nevertheless, the protocol designed for this study is practical and easily implementable for testing large samples in a wild setting, and future research might take the opportunity to further investigate the effects of a wild versus a captive environment on primate VSR, as well as to routinely include larger and more representative samples. For example, future studies could employ our mark test protocols to test samples of chimpanzees both in the wild and in captivity, comparing rates of mark-directed touching between the two groups for equal levels of mirror exposure to investigate this observation further.

While the evidence for primate VSR beyond the great apes is scanty, few studies have been conducted outside a captive setting, with large samples or with adequate controls. Here, we designed and implemented the first controlled mirror mark test on a new species in a wild setting, with a large sample size and a visible mark condition. We found that wild baboons fail to show VSR according to the standard of the mirror test, but they show some intermediate level of mirror understanding. We argue that, as is the case in other monkeys, their response is indicative of a gradualist interpretation of self-awareness across primates.

## Data Availability

The data and R code used in this paper are available on Dryad [73]. Supplementary material is available online [74].
